# Increased serum methylmalonic acid levels were associated with the presence of cardiovascular diseases

**DOI:** 10.3389/fcvm.2022.966543

**Published:** 2022-10-10

**Authors:** Xiaoya Wang, Wudi Li, Meixiang Xiang

**Affiliations:** Department of Cardiology, Second Affiliated Hospital, Zhejiang University School of Medicine, Hangzhou, China

**Keywords:** methylmalonic acid, vitamin B12, cardiovascular disease, oxidative stress, mitochondrial dysfunction

## Abstract

**Background:**

Functional vitamin B12 deficiency is common in cardiovascular diseases (CVDs), such as heart failure and myocardial infarction. Methylmalonic acid (MMA) is a specific and sensitive marker of vitamin B12 deficiency. However, there are scarce data in regard to the relationship between MMA and CVDs.

**Materials and methods:**

In this cross-sectional study, we analyzed data of 5,313 adult participants of the National Health and Nutrition Examination Survey (NHANES) 2013–2014. Associations between MMA and other variables were assessed with linear regression models. Univariable and multivariable logistic regression models were employed to explore the association between MMA and CVDs.

**Results:**

The weighted prevalence of CVDs was 8.8% in the general population of the USA. Higher MMA levels were found in participants with CVDs (*p* < 0.001). Linear regression models revealed positive associations between serum MMA level and age (*p* < 0.001), glycohemoglobin (*p* = 0.023), fasting glucose (*p* = 0.044), mean cell volume (*p* = 0.038), and hypertension (*p* = 0.003). In the multivariable logistic model adjusting for age, gender, ethnicity, smoking, hypertension, glycohemoglobin, body mass index (BMI), low-density lipoprotein-cholesterol (LDL-C), renal dysfunction and vitamin B12, serum MMA (adjusted odds ratio, 3.08; 95% confidence interval: 1.63–5.81, *p* = 0.002, per ln nmol/L increment) was associated with CVDs.

**Conclusion:**

Our study demonstrated that elevated serum MMA levels were independently associated with the presence of CVDs and may be used to predict the occurrence of CVDs.

## Introduction

Metabolic vitamin B12 deficiency is common in the general population, with a prevalence ranging between 10 and 40% (1). However, it is frequently missed and may contribute to many diseases such as Alzheimer’s disease, Parkinson’s disease (2), and stroke in older people (1). Measuring serum vitamin B12 alone is not sufficient for the diagnosis of metabolic vitamin B12 deficiency, since marginal vitamin B12 levels could be found in subclinical vitamin B12 deficiency (3). It is necessary to measure functional markers of vitamin B12 adequacy, such as serum total homocysteine (HCys) and methylmalonic acid (MMA) (1).

Vitamin B12 deficiency leads to increase of Hcys and MMA, and Hcys has been considered as an independent risk factor for cardiovascular diseases (CVDs) (4). Compared with HCys, MMA is a more specific and sensitive biomarker of subclinical vitamin B12 deficiency (5–7). Remarkably, patients with heart failure (HF) have elevated MMA, which is independent of other confounding comorbidities (8). Severe cardiomyopathy could also be found in patients with methylmalonic acidemia, an autosomal recessive disorder of metabolism (9). Moreover, MMA levels were detected to be significantly increased in patients with myocardial infarction (MI) (10), and strongly associated with cardiovascular mortality in the general population (11, 12). However, few studies have illustrated the relationship between serum MMA and the occurrence of CVDs. Therefore, in this study, we aim to explore whether elevated serum MMA levels are related to increased risk of CVDs.

## Materials and methods

### Study design and participants

National Health and Nutrition Examination Survey (NHANES), conducted by National Center for Health Statistics (NCHS), are a serial of surveys based on multistage and stratified sampling design to investigate the health status of non-institutionalized USA population of all ages. Demographic, dietary, socioeconomic and health related data were collected through home interviews. Physical and laboratory examination were done in Mobile Examination Centers (MEC). The research protocols were approved by the NCHS Research Ethics Review Board, and informed consent was provided by all participants. Information in detail is available on the NHANES website.^1^

**FIGURE 1:**
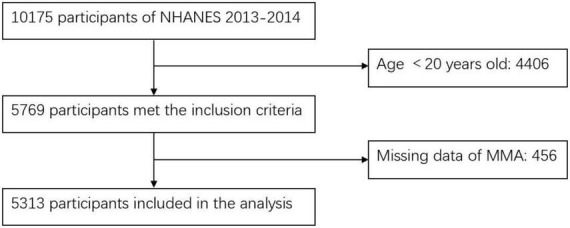
Flow chart of the study.

In this cross-sectional study, we screened 5,769 participants who aged above 20 years old and examined in the MEC from NHANES 2013–2014. Participants without data of MMA levels (*n* = 456) were excluded. Ultimately, 5,313 participants were enrolled in this study. The process of participants inclusion and exclusion was shown in Figure 1.

### Questionnaire and lab tests

General demographic variables, including age, sex, race, smoking, and alcohol consumption were collected from self-reported questionnaires. Blood samples were collected, centrifuged and stored following standardized procedures. MMA was analyzed by Liquid chromatography tandem-mass spectrometry (LC-MS/MS). All the detection protocols are available on the NHANES website.^2^

### Cardiovascular diseases assessment

Cardiovascular diseases included the self-reported diagnosis of HF, heart attack, coronary heart disease (CHD), angina pectoris, and stroke. The diagnosis was confirmed in the NHANES through asking questions whether participants “were told by a doctor or other health care providers” about one of the CVDs.

### Statistical analysis

Continuous variables were presented as mean ± standard deviation (SD), and compared among participants with/without CVDs using Student’s *t*-test or Mann–Whitney *U* test. Categorical demographic variables were presented as numbers (weighted percentage) and compared among participants with/without CVDs using Chi-square tests. For further analyses, variables with skewed distributions were ln-transformed to normalize their distributions.

Relationships between MMA and covariates were examined through univariable and multivariable linear regression models. Univariable and multivariable logistic regression models were employed to explore the associations between MMA and the presence of CVDs and individual types of CVDs (HF, CHD, MI, stroke, and angina pectoris). Confounding variables, age, gender, and ethnicity were included in the models.

All analyses were performed according to the guidelines set by the Centers for Disease Control and Prevention for analysis of NHANES dataset. Complex samples module in SPSS version 22.0 (IMB Corp., Armonk, New York, USA) was used to adjust for the clustered hierarchical sample designs of NHANES, using cluster, stratum, and sample weights provided by NCHS. All statistical tests were two-tailed, and a *p*-value less than 0.05 denoted the level of significance.

## Results

### Population characteristics

Table 1 presents the characteristics of the weighted sample included in the study. The sample included a higher percentage of female (51.7%) than male (48.3%). The weighted prevalence of CVDs was 8.8% in the general population. Higher MMA levels were found in participants with CVDs (229.83 ± 165.32 vs. 169.90 ± 160.19 nmol/L, *p* < 0.001). Significant differences were also found in other variables including age, ethnicity, smoking, diabetes, hypertension, body mass index (BMI), lipid profiles, renal dysfunction, vitamin B12, folate, and hemoglobin (all *p* < 0.05).

**TABLE 1:** Population characteristics stratified by CVDs.

Characteristic	Overall *n* = 5313	CVD *n* = 547	Non-CVD *n* = 4766	*P*-value
**Sociodemographic**				
Age, year	49.09 ± 17.50	65.93 ± 12.85	47.16 ± 16.91	< **0.001**
Gender				0.247
Male	2534 (48.3%)	299 (52.8%)	2235 (47.8%)	
Female	2779 (51.7%)	248 (47.2%)	2531 (52.2%)	
Ethnicity				< **0.001**
Mexican American	720 (9.2%)	51 (5.1%)	699 (9.6%)	
Other Hispanic	470 (5.5%)	36 (3.3%)	434 (5.8%)	
Non-Hispanic White	2317 (66.5%)	307 (76.7%)	2010 (65.5%)	
Non-Hispanic Black	1044 (10.8%)	110 (10.1%)	934 (10.9%)	
Non-Hispanic Asian	604 (5.3%)	33 (3.0%)	571 (5.5%)	
Other race – including multi-racial	158 (2.7%)	10 (1.9%)	148 (2.8%)	
**Personal history**				
Smoking	2312 (43.5%)	319 (59.0%)	1993 (42.0%)	< **0.001**
Drinking	3519 (77.2%)	358 (74.2%)	3161 (77.5%)	0.301
**Self-reported medical conditions**				
Diabetes	663 (10.1%)	179 (29.4%)	484 (8.2%)	< **0.001**
Hypertension	1978 (34.9%)	401 (69.9%)	1577 (31.5%)	< **0.001**
Hyperlipidemia	1880 (35.4%)	350 (65.1%)	1530 (32.5%)	< **0.001**
Renal dysfunction	171 (2.5%)	67 (10.1%)	104 (1.8%)	< **0.001**
**Physical examination**				
BMI, kg/m^2^	29.13 ± 7.11	30.34 ± 7.91	28.99 ± 7.00	< **0.001**
**Laboratory test**				
MMA, nmol/L	176.07 ± 161.74	229.83 ± 165.32	169.90 ± 160.19	< **0.001**
Vitamin B12, pmol/L	471.93 ± 554.07	538.42 ± 599.70	464.31 ± 548.15	**0.035**
RBC folate, nmol/L	1222.81 ± 569.10	1454.22 ± 738.45	1196.42 ± 540.30	< **0.001**
Serum total folate, nmol/L	45.32 ± 33.13	58.10 ± 71.7	43.85 ± 24.77	< **0.001**
Total cholesterol, mmol/L	4.89 ± 1.08	4.54 ± 1.09	4.93 ± 1.07	< **0.001**
HDL-C, mmol/L	1.37 ± 0.42	1.31 ± 0.44	1.37 ± 0.41	**0.002**
LDL-C, mmol/L	2.87 ± 0.91	2.46 ± 0.88	2.92 ± 0.90	< **0.001**
Triglycerides, mmol/L	1.37 ± 1.41	1.45 ± 0.92	1.35 ± 1.46	0.287
Glycohemoglobin, %	5.74 ± 1.07	6.21 ± 1.24	5.68 ± 1.04	< **0.001**
Fasting glucose, mmol/L	5.97 ± 1.92	6.65 ± 2.18	5.89 ± 1.87	< **0.001**
Insulin, pmol/L	80.10 ± 119.02	109.27 ± 151.87	76.67 ± 114.10	< **0.001**
Hemoglobin, g/dL	13.98 ± 1.53	13.69 ± 1.69	14.02 ± 1.51	< **0.001**
Mean cell volume, fL	89.19 ± 5.97	90.32 ± 6.32	89.06 ± 5.91	< **0.001**
Mean cell hemoglobin, pg	30.16 ± 2.50	30.49 ± 3.22	30.12 ± 2.40	**0.004**
MCHC, g/dL	33.79 ± 1.10	33.73 ± 1.89	33.80 ± 0.97	**0.001**
Red cell distribution width, %	13.73 ± 1.27	14.34 ± 1.56	13.66 ± 1.22	< **0.001**

Continuous variables were presented as mean ± SD, categorial variables were presented as *n* (weighted percentage). CVD, cardiovascular disease; BMI, body mass index; MMA, methylmalonic acid; RBC, red blood cell; HDL-C, high-density lipoprotein-cholesterol; LDL-C, low-density lipoprotein-cholesterol; MCHC, mean corpuscular hemoglobin concentration. Bold values are *p* < 0.05.

### Association of methylmalonic acid with covariates

The association of MMA with other covariates was shown in Supplementary Tables 1, 2. In univariate linear regression analysis, serum MMA level was positively associated with age, glycohemoglobin, fasting glucose, and mean cell volume (all *P* < 0.05), while negative association was found between serum MMA level and vitamin B12 (*p* < 0.05). Higher MMA level existed in non-Hispanic White, smokers, and participants with diabetes, hypertension, hyperlipidemia and renal dysfunction (all *p* < 0.05). Multivariate linear regression, which included every significant variable detected by univariate linear regression, identified age, ethnicity, hypertension, vitamin B12, glycohemoglobin, fasting glucose, and mean cell volume as independent determinants of serum MMA level.

### Association of serum methylmalonic acid level and cardiovascular diseases

Tables 2, 3 present the association between serum MMA level and the presence of CVDs. In the univariate model, the odds ratio (OR) of MMA (per ln nmol/L increase) for total CVDs was 2.93 [95% confidence interval (CI): 2.38–3.61, *p* < 0.001]. In adjusted model 1, after adjusting for the basic demographic variables, including age, gender and ethnicity, the OR of MMA for total CVDs was 1.56 (95% CI: 1.22–2.00, *p* = 0.002). In adjusted model 2, after additionally adjusting for the traditional CVDs risk factors, including smoking, hypertension, Glycohemoglobin, BMI and low-density lipoprotein-cholesterol (LDL-C), the OR of MMA for total CVDs was 1.48 (95% CI: 1.09–2.01, *p* < 0.05). In adjusted model 3, after further adjusting for renal dysfunction and vitamin B12 which has been demonstrated to be associated with MMA in the previous studies (13, 14) and this study, the OR of MMA for total CVDs was 3.08 (95% CI: 1.63–5.81, *P* = 0.002). For specific types of CVDs, significant associations existed between MMA and HF, CHD, angina pectoris, heart attack, and stroke in univariable models (all *p* < 0.001). After adjusting for all model 3 variables, only HF had a significant association with MMA level (95% CI: 1.45, 3.33, *P* = 0.001).

**TABLE 2:** Associations between MMA (ln nmol/L) and CVDs by univariable logistic regression model.

Outcome	OR^a^ (95% CI)	*P*-value
Total CVDs	2.93 (2.38, 3.61)	<**0.001**
Heart failure	2.82 (2.17, 3.68)	<**0.001**
Coronary heart disease	2.51 (1.97, 3.22)	<**0.001**
Angina pectoris	2.13 (1.71, 2.64)	<**0.001**
Heart attack	2.98 (2.24, 3.95)	<**0.001**
Stroke	3.12 (2.21, 4.42)	<**0.001**

*^a^*Odds ratio of MMA, per ln nmol/L increase.

OR, odds ratio; CI, confidence interval; MMA, methylmalonic acid; CVDs, cardiovascular diseases. Bold values are *p* < 0.05.

**TABLE 3:** Associations between MMA (ln nmol/L) and CVDs by multivariable logistic regression model.

Outcome	Adjusted model 1^a^		Adjusted model 2^b^		Adjusted model 3^c^	
	OR^d^ (95% CI)	*P*-value	OR (95% CI)	*P*-value	OR (95% CI)	*P*-value
Total CVDs	1.56 (1.22, 2.00)	**0.002**	1.48 (1.09, 2.01)	**0.016**	3.08 (1.63, 5.81)	**0.002**
Heart failure	1.61 (1.21, 2.13)	**0.003**	1.62 (1.25, 2.11)	**0.001**	2.19 (1.45, 3.33)	**0.001**
Coronary heart disease	1.23 (0.95, 1.58)	0.111	1.11 (0.93, 1.31)	0.228	1.18 (0.17, 8.37)	0.861
Angina pectoris	1.10 (0.78, 1.55)	0.577	1.19 (0.76, 1.86)	0.414	1.22 (0.44, 3.40)	0.688
Heart attack	1.75 (1.14, 2.68)	**0.015**	1.28 (1.00, 1.64)	**0.049**	2.34 (0.12, 46.66)	0.553
Stroke	1.81 (1.02, 3.20)	**0.042**	0.96 (0.65, 1.41)	0.804	2.37 (0.30, 18.47)	0.386

*^a^*Adjusted Model 1: this model was adjusted for age, gender, and ethnicity.

*^b^*Adjusted Model 2: additionally adjusted for smoking, hypertension, Glycohemoglobin, BMI, LDL-C.

*^c^*Adjusted Model 3: additionally adjusted for renal dysfunction, vitamin B12.

*^d^*Odds ratio of MMA, per ln nmol/L increase.

OR, odds ratio; CI, confidence interval; MMA, methylmalonic acid; CVDs, cardiovascular diseases; BMI, body mass index; LDL-C, low-density lipoprotein-cholesterol. Bold values are *p* < 0.05.

## Discussions

In this large cross-sectional study of 5,313 adults recruited from a nationally representative sample of the USA, we found that increasing serum MMA levels were significantly associated with the presence of CVDs, even in the absence of low serum vitamin B12 concentration.

Vitamin B12 plays an important role in cellular metabolism, especially in mitochondrial metabolism, methylation, and DNA synthesis (15). As an active superoxide scavenger, reduced forms of vitamin B12 are indispensable for its coenzyme activity (16). However, only 25% of the serum total vitamin B12 is in the active form of holotranscobalamin (17). Additionally, oxidative byproducts could impair cellular uptake of vitamin B12, which leads to functional vitamin B12 deficiency (13). Therefore, total serum vitamin B12 levels are not sensitive for vitamin B12 deficiency.

Methylmalonic acid is derived from the hydrolysis of D-methylmalonyl-CoA (MMA-CoA). Normally, MMA-CoA is converted to succinyl-CoA and involved in the tricarboxylic acid cycle (TCA) with vitamin B12 as a cofactor (8). Oxidative byproducts may prevent cellular utilization of vitamin B12, and a deficiency of vitamin B12 at the tissue level leads to increase of MMA before serum vitamin B12 decreases (18). MMA level is also a marker of mitochondrial dysfunction. Metabolism of MMA-CoA occurs in healthy mitochondria, and mitochondria insufficiency contributes to MMA accumulation which further impacts on mitochondrial electron transport chain (ETC) and redox status (15, 19). In genetic methylmalonic acidemia, ETC dysfunction induced by MMA accumulation has been considered to be the major pathophysiology to cause muscular hypotonia and progressive neurological deterioration (19–22). Experimental studies revealed that decreased amounts and activity of the TCA enzymes existed in mice with methylmalonic acidemia (23), and MMA could competitively inhibit state 3 mitochondrial respiration (24). Therefore, MMA accumulation in certain conditions may not be caused by low vitamin B12 concentration, but associated with oxidative stress and mitochondrial dysfunction, which may explain high levels of both vitamin B12 and MMA in patients with clinical comorbidities.

A cross-sectional clinical study demonstrated that compared to healthy controls, MMA levels were remarkably increased in 43.8% of patients with HF (8). MMA levels were also significantly higher in cases with MI, whereas vitamin B12 showed no clear association with MI (10). One study (8) revealed that a significant negative correlation between MMA and vitamin B12 levels only presented in patients without comorbidities such as right heart failure, heart failure with reduced ejection fraction, hypertension, atrial fibrillation, thyroid disease and kidney disease. The correlation became progressively less negative if two or more comorbidities were present. When more than 4 clinical conditions coexisted, MMA levels correlated positively with vitamin B12 levels, with increased MMA at increased vitamin B12 levels. Same to the previous research, our study showed that CVDs patients who were more likely to have comorbidities such as hypertension and renal dysfunction, had higher levels of both vitamin B12 and MMA.

Oxidative stress is known to occur in various types of CVDs. An increase in the formation of reactive oxygen species (ROS) and/or a decrease in the antioxidant reserve, contributes to oxidative stress in cardiac and vascular myocytes. Factors, such as the activation of renin-angiotensin and sympathetic system, as well as endothelial dysfunction, promote oxidative stress and subsequent cardiovascular tissue injury. Consequently, vascular defects cause hypertension and atherosclerosis, and cardiac defects lead to contractile dysfunction and HF (25). Mitochondrial function is also essential for vascular cell growth and function. Mitochondrial dysfunction impairs energy production and cell physiology, which produces apoptosis-, oxidative- and calcium mediated myocyte injury. Mitochondria dysfunction plays an important role in cardiac ischemia, atherosclerotic plaque rupture and HF (26, 27).

Consistent with previous findings (14, 28, 29), we found that MMA levels increased in participants with oxidative risks, including smoking, diabetes, hypertension, hyperlipidemia, renal dysfunction, and aging. This study further revealed that elevated MMA levels were related to the presence of CVDs, which was independent of demographic and classic cardiovascular risk factors. After adjustment of renal dysfunction and vitamin B12, the association was even more significant, suggesting that functional vitamin B12 deficiency may be more related to CVDs. It would be of clinical significance to use MMA to predict the development of CVDs. Besides, a large prospective cohort study showed that MMA level at baseline was strongly associated with all-cause and cardiovascular mortality in the general population (12).

It has been demonstrated that vitamin B12 supplementation could reduce the elevated serum levels of both MMA and Hcys in end-stage renal disease (ESRD) patients with low vitamin B12 levels (30). Oral B-vitamin therapy with folic acid, vitamin B12 and vitamin B6, is effective to normalize MMA and Hcys in apparently healthy elderly subjects (31). Using elevated MMA to identify patients with clinically relevant metabolic deficiency and supplementing vitamin B12 in these patients with polyneuropathy has been proved to improve or stabilize symptoms (32). Thus, vitamin B12 may be supplemented to lower MMA and slow the development of CVDs in patients with elevated MMA. Previous studies failed to demonstrate that the regular administration of B vitamins reduces CVDs risk despite the lowering of Hcys (33–35). However, in these studies, vitamin B was administered indiscriminately without considering the baseline levels of Hcys, MMA or vitamin B12. What’s more, some clinical trials (36, 37) even found that elevated vitamin B12 was associated with higher mortality. Therefore, it is reasonable to supplement vitamin B12 in CVDs patients with higher MMA without significantly elevated vitamin B12. For CVDs patients with both higher MMA and vitamin B12 levels, antioxidant drugs such as vitamin A, C, and E may be used. Although one systemic review (38) revealed that vitamin and mineral supplementation provided little or no benefit in preventing CVDs, trials included in the review did not monitor the instant treatment response to guide the use of antioxidants. A fine balance between the presence of ROS and antioxidants is important for the proper normal functioning of the cell (39), and indiscriminate use of antioxidants may be harmful (40). A large randomized trial of vitamin E supplementation (41) showed that 600 IU of vitamin E every other day for 10 years did not provide significant benefits on major cardiovascular events in the general healthy women, but there was a significant 26% reduction in major cardiovascular events among the subgroup of women aged more than 65 years. Serum MMA measurement, which is feasible in the clinical situation, could be used to guide the application of antioxidant supplements, thereby identifying subgroup patients in which CVDs outcome may improve.

Hence, our study showed that higher serum MMA was associated with the presence of CVDs, and the connection between MMA and CVDs may be related to oxidative stress and mitochondrial dysfunction. Serum MMA should be measured in patients at risk of vitamin B12 deficiency, as well as with oxidative risks. It has the potential to use serum MMA to predict the occurrence of CVDs and guide the treatment targeting oxidative stress and subclinical vitamin B12 deficiency.

### Study strength and limitations

To the best of our knowledge, this is the first study exploring the association between serum MMA levels and CVDs in a nationally representative sample. The strength of our study is the large sample size and the application of complex samples module in the SPSS to adjust for the clustered hierarchical sample designs of NHANES, which has made the result more convincing.

This study also has several limitations. First, as a cross-sectional study, a causal relationship between MMA and CVDs could not be concluded; thus, further cohort studies are needed to confirm the association. Second, inborn and postnatal increase of MMA could not be distinguished. Nevertheless, the incidence of hereditary methylmalonic acidemia was very low in the general population, therefore it is unlikely to alter the result. Third, same to the previous studies (42, 43), CVDs were identified by self-reporting, which may elicit recall and interviewer bias. But the diagnosis of CVDs was consistent throughout the study. As described on the NHANES website (see text footnote 1), after the question such as “has a doctor or other health professional ever told you that you had a heart attack,” the participants were required to specify the certain age when he or she was told to have a heart attack. In this regard, the answers were reconfirmed. Moreover, questions evaluating cardiovascular health were also asked in other sections of the questionnaire and all MEC interview data were keyed directly into an automated data entry system with built-in error and consistency checks. Besides, all the MEC interviewers were well trained to administer the MEC Questionnaires in a standardized fashion to guarantee the consistency and accuracy of information. There was also home office who would evaluate completed MEC interviews to look for problems to ensure quality. All these processes minimize the recall and interviewer bias to the greatest extent.

## Conclusion

Cardiovascular diseases were common in the general population of the USA, and higher serum MMA levels were found in participants with CVDs. Besides, serum MMA levels were positively correlated to age, glycohemoglobin, fasting glucose, mean cell volume and hypertension. Moreover, increased serum MMA levels were associated with the presence of CVDs, which was independent of demographic and classic CVDs risk factors. The relationship between MMA and CVDs may be related to oxidative stress and mitochondrial dysfunction. Serum MMA levels could potentially be used to predict the occurrence of CVDs, but further studies are needed to investigate and clarify it.

## Data availability statement

Publicly available datasets were analyzed in this study. This data can be found here: https://www.cdc.gov/nchs/nhanes/.

## Ethics statement

Ethical review and approval was not required for the study on human participants in accordance with the local legislation and institutional requirements. Written informed consent for participation was not required for this study in accordance with the national legislation and the institutional requirements.

## Author contributions

XW and WL conceptualized and wrote the manuscript. MX conceptualized, reviewed, and modified the manuscript. All authors approved the final version of the manuscript.

## Footnotes

1
https://www.cdc.gov/nchs/nhanes/

2
https://wwwn.cdc.gov/nchs/nhanes/continuousnhanes/labmethods.aspx?BeginYear=2013
