# Supplementary material for: Increased serum methylmalonic acid levels were associated with the presence of cardiovascular diseases
Source: Front Cardiovasc Med. 2022 Oct 10;9:966543. doi: 10.3389/fcvm.2022.966543 (PMC9588910; doi:10.3389/fcvm.2022.966543)
Supplement: Supplementary file 1 [file Data_Sheet_1.pdf]

Supplementary Material

Supplementary Table S1 Associations between MMA (In nmol/L) and Other Covariates by Univariable Linear Regression Model

**Characteristic**	**Beta**	**95% CI**	***p*-value**
**Sociodemographic**			
Age, year	0.009	0.008, 0.010	＜0.001
Gender			
Male	0.005	-0.032, 0.041	0.787
Female	Ref	Ref	Ref
Ethnicity			
Mexican American	Ref	Ref	Ref
Other Hispanic	0.087	0.019, 0.156	0.016
Non-Hispanic White	0.290	0.237, 0.343	＜0.001
Non-Hispanic Black	0.055	-0.001, 0.111	0.055
Non-Hispanic Asian	0.112	0.045, 0.179	0.003
Other Race - Including Multi-Racial	0.196	0.119, 0.272	＜0.001
**Personal History**			
Smoking	0.058	0.019, 0.096	0.006
Drinking	-0.020	-0.060， 0.020	0.306
**Self-reported Medical Conditions**			
Diabetes	0.044	0.019, 0.068	0.002

Hypertension	0.151	0.122, 0.179	<0.001
Hyperlipidemia	0.059	0.047, 0.072	<0.001
Renal dysfunction	0.111	0.014, 0.208	0.028
**Physical Examination**			
BMI, kg/m2	-0.001	-0.003, 0.002	0.598
**Laboratory Test**			
Vitamin B12, ln pmol/L	-0.230	-0.255, -0.204	<0.001
RBC folate, ln nmol/L	0.045	0.002, 0.088	0.043
Serum total folate, ln nmol/L	-0.002	-0.025, 0.021	0.853
Total cholesterol, mmol/L	-0.003	-0.022, 0.015	0.701
HDL-C, mmol/L	0.001	-0.045, 0.048	0.952
LDL-C, mmol/L	-0.009	-0.034, 0.017	0.483
Triglycerides, ln mmol/L	0.032	-0.013, 0.076	0.151
Glycohemoglobin, %	0.044	0.024, 0.064	<0.001
Fasting glucose, ln mmol/L	0.157	0.047, 0.266	0.008
Insulin, ln pmol/L	0.015	-0.019, 0.049	0.368
Hemoglobin, g/dL	-0.014	-0.029, 0.002	0.080
Mean cell volume, fL	0.008	0.006, 0.011	<0.001
Mean cell hemoglobin, pg	0.016	0.007, 0.025	0.001
MCHC, g/dL	0.006	-0.014, 0.025	0.558
Red cell distribution width, %	0.016	-0.002, 0.035	0.076

CI, confidence interval; Ref, reference; BMI, body mass index; RBC, red blood cell; HDL-C, high-density lipoprotein-cholesterol; LDL-C, low-density lipoprotein-cholesterol; MCHC, mean corpuscular hemoglobin concentration.

Supplementary Table S2 Associations between MMA (In nmol/L) and Other Covariates by Multivariable Linear Regression Model

**Characteristic**	**Beta**	**95% CI**	***p*-value**
Sociodemographic			
Age, year	0.007	0.005, 0.009	＜0.001
Ethnicity			
Non-Hispanic White	0.152	0.088, 0.216	＜0.001
Self-reported Medical Conditions			
Hypertension	0.060	0.023, 0.096	0.003
Laboratory Test			
Vitamin B12, ln pmol/L	-0.259	-0.311, -0.206	＜0.001
Glycohemoglobin, %	0.067	0.011, 0.123	0.023
Fasting glucose, ln mmol/L	-0.268	-0.527, -0.008	0.044
Mean cell volume, fL	0.049	0.003, 0.095	0.038

CI, confidence interval
